# Optimization of the fermentation culture conditions of *Bacillus amyloliquefaciens* ck-05 using response surface methodology

**DOI:** 10.3389/fmicb.2025.1552645

**Published:** 2025-03-12

**Authors:** Xiaoyu Liu, Jerome Jeyakumar John Martin, Xinyu Li, Lixia Zhou, Rui Li, Qihong Li, Jianwei Zhang, Dengqiang Fu, Hongxing Cao

**Affiliations:** ^1^Coconut Research Institute, Chinese Academy of Tropical Agricultural Sciences, Wenchang, China; ^2^National Key Laboratory for Tropical Crop Breeding, Chinese Academy of Tropical Agricultural Sciences, Haikou, China; ^3^Institute of Apicultural Research, Chinese Academy of Agricultural Sciences, Beijing, China

**Keywords:** *Bacillus amyloliquefaciens* ck-05, culture conditions, optimization, response surface methodology, fermentation optimization

## Abstract

*Bacillus amyloliquefaciens* is widely recognized for its potential as a biofertilizer and biocontrol agent in agriculture due to its plant growth-promoting (PGP) mechanisms. However, the practical application of this bacterium is often limited by suboptimal fermentation conditions, which hinder its growth and efficacy. While numerous studies have optimized growth conditions for various strains of *B. amyloliquefaciens*, the novelty of this work lies in the systematic optimization of fermentation conditions for *B. amyloliquefaciens* ck-05, a strain obtained from a culture collection, and its potential application as a biofertilizer. In this study, single-factor experiments were conducted to evaluate the effects of carbon and nitrogen sources, inorganic salts, pH, temperature, culture time, rotation speed, inoculation rate, and liquid volume on the OD600 value of strain ck-05. A Plackett-Burman design was used to identify the significant factors influencing OD600, followed by a Box-Behnken design to determine the optimal growth conditions. The results revealed that soluble starch, peptone, and magnesium sulfate significantly impacted the growth of *B. amyloliquefaciens* ck-05. The optimized fermentation conditions were determined to be pH 6.6, temperature 30°C, culture time 40 h, rotation speed 150 rpm, inoculum rate 0.8%, and liquid volume 40%. Post-optimization, the OD600 of the fermentation broth increased by 72.79% compared to pre-optimization levels. The culture and fermentation conditions for *B. amyloliquefaciens* ck-05 were successfully optimized, providing a theoretical foundation for the future development of this strain as a microbial fertilizer.

## 1 Introduction

In the development of high-efficiency agriculture, chemical fertilizers have played a significant role in increasing crop yields. However, the widespread practice of excessive and indiscriminate use of chemical fertilizers in pursuit of higher yields has led to numerous issues, including soil acidification, compaction, nutrient imbalances, and agricultural non-point source pollution. Microbial fertilizers, a new generation of fertilizers introduced in the 21st century, offer a sustainable alternative. Long-term application of microbial fertilizers can enhance soil fertility, improve the soil micro-ecological environment, and promote crop growth.

*Bacillus amyloliquefaciens* is a well-studied plant growth-promoting rhizobacterium belonging to the *Bacillus* genus, which is widely distributed in nature. It possesses desirable traits such as efficient spore production, excellent thermal stability, strong stress resistance, and rapid growth and reproduction. These characteristics make *B. amyloliquefaciens* a promising candidate for use as a microbial fertilizer. Successful applications of *B. amyloliquefaciens* in promoting crop growth have already been documented (Qin et al., 2017; Chen et al., 2016).

However, different microorganisms have unique growth, reproduction, and metabolic processes, which result in varying nutritional and environmental requirements. Optimizing the fermentation medium and culture conditions for growth-promoting strains from different sources is essential for the successful development and application of growth-promoting agents (Ahsan et al., 2017). *B. amyloliquefaciens* ck-05, a strain with the potential to be developed into a microbial fertilizer, was identified in the early stages of our research group’s work (Liu et al., 2023). Therefore, optimizing the fermentation culture conditions for *B. amyloliquefaciens* ck-05 is particularly important. However, to date, no studies have been conducted to optimize the fermentation conditions for this strain.

The response surface optimization method is one of the most widely used approaches for experimental design and statistical analysis in the field of microbial culture (Jin, 2014; Wang et al., 2016). Compared to single-factor and orthogonal tests, this method is more comprehensive, significantly reduces the number of experiments required, and identifies optimal fermentation conditions with less experimental data. It not only minimizes the workload involved in fermentation optimization but also enhances the accuracy and rationality of the process (Guo et al., 2020; Priyadharshini and Bakthavatsalam, 2016). For example, the response surface method was used to optimize the fermentation conditions of the alginate-degrading *Cobetia* sp. 20, resulting in a 26.36% increase in enzyme activity for the optimized strain (Tang et al., 2024).

This study identified the key factors influencing the growth of strain ck-05 from various parameters, including medium formulation and culture conditions, using the Plackett-Burman (PB) test. The central points of the Box-Behnken Design (BBD) were determined through the steepest ascent test. Ultimately, the optimal medium formulation and nutritional conditions were established using the response surface design method (Li et al., 2021; Lu et al., 2020).

Through optimization experiments, the OD600 value of the fermentation broth of strain ck-05 was significantly improved. This provides both a theoretical foundation and experimental data for the development of *Bacillus amyloliquefaciens* ck-05 as a microbial fertilizer for production and application.

## 2 Materials and methods

### 2.1 Microorganisms

Test Strains: *Bacillus amyloliquefaciens* ck-05 was isolated and screened by our research group and is currently stored at the Preservation and Management Center of Chinese General Microbial Strains under the preservation number CGMCC19565.

### 2.2 Media

• LB (Luria-Bertan) Medium: Peptone 10 g, sodium chloride 10 g, yeast extract 5 g, agar 20 g, water 1 l, pH 7.2 ± 0.2.

• Basic Fermentation Medium: Glucose 15 g, peptone 15 g, dipotassium hydrogen phosphate trihydrate 3 g, magnesium sulfate heptahydrate 0.5 g, calcium chloride 0.1 g, distilled water 1 I, pH 7.2 ± 0.2.

### 2.3 Optimization of media

#### 2.3.1 Microorganism activation

For activation of microorganisms from the glycerol stock, the standard protocol was followed. A small volume of the bacterial suspension was aseptically removed from the 30% glycerol stock stored at −80°C and inoculated into a sterile triangular flask containing LB liquid medium. The culture was incubated at 30°C with shaking at 200 rpm for 24–48 h to allow for proper recovery and growth. After incubation, 0.1 mL of the broth culture was streaked onto LB agar plates and incubated at 30°C for 24–48 h to check for purity.

#### 2.3.2 Seed solution preparation

A single colony of strain ck-05 was inoculated into an Erlenmeyer flask containing LB liquid medium and cultured at 30°C with a rotation speed of 200 rpm for 48 h. The prepared seed solution was set aside for subsequent experiments.

#### 2.3.3 Initial culture conditions

The strain was cultured in the basic fermentation medium for 48 h with 40% liquid volume in the flask, 1% inoculation amount, 30°C, and a rotation speed of 200 rpm. The OD600 of the seed culture used for inoculation was 1.415.

#### 2.3.4 Detection method

The optical density at 600 nm (OD600) of the fermentation broth was measured using a microplate reader, with the blank medium serving as the control.

#### 2.3.5 Growth curve determination method

Strain ck-05 was inoculated into LB liquid medium with a 1% inoculation amount and cultured at 30°C with a rotation speed of 200 rpm. The OD600 value was recorded every 2 h to determine the growth curve.

#### 2.3.6 Single-factor optimization experiment

Optimization of Medium Components: The optimal carbon source, nitrogen source, and inorganic salt were identified following the methods used in previous studies (Hong et al., 2013; Ye et al., 2011). Various carbon sources, including glucose, sucrose, fructose, lactose, mannitol, soluble starch, and maltose, were tested. Nitrogen sources such as peptone, yeast extract, tryptone, urea, ammonium nitrate, ammonium chloride, and ammonium sulfate were evaluated. Inorganic salts tested included magnesium sulfate, calcium chloride, calcium carbonate, dipotassium hydrogen phosphate, sodium chloride, manganese sulfate, and ferrous sulfate, each replacing the corresponding component in the basic medium. The OD600 value was measured after culturing under the initial culture conditions to determine the optimal carbon source, nitrogen source, and inorganic salt.

#### 2.3.7 Optimization of culture conditions

Based on the screening results for carbon source, nitrogen source, and inorganic salt, an optimized medium was used to investigate fermentation conditions. Factors evaluated included fermentation temperature, initial inoculation rate, pH, liquid volume, and rotational speed (Shi et al., 2023). The inoculation rates tested ranged from 0.5%, 1.0%, 2.0%, 3.0%, 4.0%, to 5.0%. The initial pH values tested ranged from 5.7, 6.0, 6.3, 6.6, 7.2, 7.5, 7.8, to 8.1. Liquid volumes were tested at 20%, 40%, 60%, 80%, and 100%. Temperatures evaluated were 25°C, 30°C, 35°C, 40°C, and 45°C. Rotational speeds ranged from 150, 175, 200, 22 to 250 rpm.

### 2.4 Response surface optimization test

Response surface methodology (RSM) is a widely used statistical approach for optimizing complex processes by modeling and analyzing the relationship between multiple independent variables and a response variable. In this study, RSM was employed to optimize the fermentation conditions for Bacillus amyloliquefaciens CK-05, with the optical density (OD600) of the fermentation broth used as the response variable. The optimization process was carried out in three sequential steps: (1) a Plackett-Burman (PB) design experiment to identify significant factors, (2) a steepest climbing test to approach the optimal range of these factors, and (3) a Box-Behnken design to model and refine the optimal conditions. Each step is described in detail below.

#### 2.4.1 Plackett-Burman (PB) design experiment

Based on the results of the single-factor optimization tests, a PB design experiment was developed using Design Expert 13.0. Each variable was assigned a low and high level, and each group was repeated three times. The OD600 value of the fermentation broth of strain ck-05 was used as the response variable to identify significant factors (Li et al., 2017; Zhang, 2006).

#### 2.4.2 Steepest climbing test

The steepest climbing test was conducted based on the significant factors identified from the PB test results. The direction and step size of changes were determined according to the effect coefficient of each factor and practical considerations. This process was used to determine the central points for the Box-Behnken design.

#### 2.4.3 Box-Behnken design

Using the variables and concentrations selected from the PB design experiment and the steepest climbing test, a Box-Behnken design was developed. The test results were analyzed using Design Expert 13.0.

### 2.5 Data processing and analysis

Data processing and graph plotting were performed using WPS Table. Statistical analyses were conducted using SAS software, and the response surface plots were generated with Design Expert 13.0.

## 3 Results and analysis

### 3.1 Growth curve of strain ck-05

**FIGURE 1:**
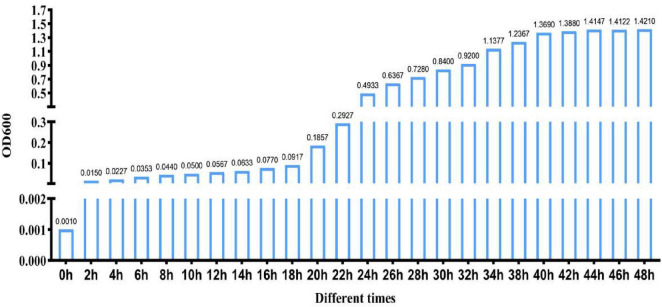
Growth curve of *Bacillus amyloliquefaciens* ck-05 in Luria-Bertan (LB) medium. The growth was monitored by measuring the OD600 at regular intervals over 48 h to assess bacterial proliferation under the given culture conditions.

Strain CK-05 was cultured in LB broth under aerobic conditions of 30°C and agitation at 150 rpm. The fermentation process was carried out continuously for 48 h, with the optical density (OD600) measured every 2 h to monitor bacterial growth (Figure 1). The growth curve of strain CK-05 revealed three distinct phases: (1) a lag phase during the first 0–18 h, characterized by slow growth as the bacteria adapted to the medium; (2) a logarithmic growth phase between 18 and 40 h, during which the bacterial population increased exponentially; and (3) a stable growth phase from 40 to 48 h, where growth plateaued as the culture reached stationary phase. Based on these observations, 40 h was determined to be the optimal culture time for strain CK-05, as it represents the end of the logarithmic phase, where bacterial biomass is maximized.

### 3.2 Single-factor optimization

#### 3.2.1 Screening of carbon source composition and concentration

**FIGURE 2:**
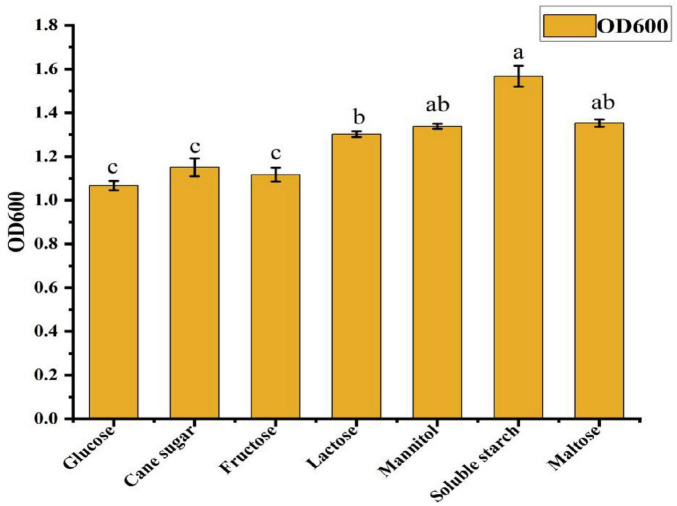
Effects of different carbon sources on the growth of *Bacillus amyloliquefaciens* ck-05. Growth values are expressed relative to the control, and data are presented as mean ± standard deviation from three independent experiments. Statistical significance was determined by analysis of variance (ANOVA), with *p*-values (*p* < 0.05) indicating significant differences among carbon sources. Error bars represent the standard deviation.

As shown in Figure 2, different carbon sources had varying effects on the cell density in the ck-05 fermentation broth. Bacterial growth was highest when soluble starch was used as the carbon source, followed by maltose, mannitol, lactose, sucrose, fructose, and glucose. The significantly higher bacterial growth observed with soluble starch compared to other carbon sources confirmed its suitability as the optimal carbon source for the fermentation culture of strain CK-05. To determine the optimal concentration of soluble starch, it was added to the basic medium at concentrations of 5, 10, 15, 20, 25, and 30 g/L. As shown in Table 1, bacterial growth peaked at a soluble starch concentration of 15 g/L, where the absorbance at 600 nm reached 1.34 ± 0.06. This represented a 12.7% increase in growth compared to the next best concentration (10 and 20 g/L, OD600 = 1.23 ± 0.05) and a 27.6% increase compared to the lowest concentration (30 g/L, OD600 = 1.05 ± 0.02). The growth at 15 g/L was significantly higher than all other concentrations (*p* < 0.05), confirming its effectiveness in promoting bacterial growth.

**TABLE:** 

Concentration	Glucose	Cane sugar	Fructose	Lactose	Mannitol	Soluble starch	Maltose
OD600	1.07c	1.15c	1.11c	1.30b	1.33ab	1.57a	1.35ab

Values represent the mean ± standard deviation (*n* = 3) of triplicate measurements. Different letters (a, b) indicate statistically significant differences between peptone concentrations (*p* < 0.05), based on a one-way analysis of variance (ANOVA).

**TABLE 1:** Effect of different concentrations of soluble starch on the bacterial growth of strain ck-05.

Concentration	5 g/L	10 g/L	15 g/L	20 g/L	25 g/L	30 g/L
OD600	1.18 ± 0.01c	1.23 ± 0.05b	1.34 ± 0.06a	1.23 ± 0.05b	1.10 ± 0.05c	1.05 ± 0.02c

The OD600 values represent the bacterial growth, measured after fermentation with various concentrations of soluble starch (5, 10, 15, 20, 25, and 30 g/L). Letters (a, b, c) indicate statistically significant differences between groups, as determined by analysis of variance (ANOVA) test. Data are presented as mean ± standard deviation.

#### 3.2.2 Screening of nitrogen source composition and concentration

**FIGURE 3:**
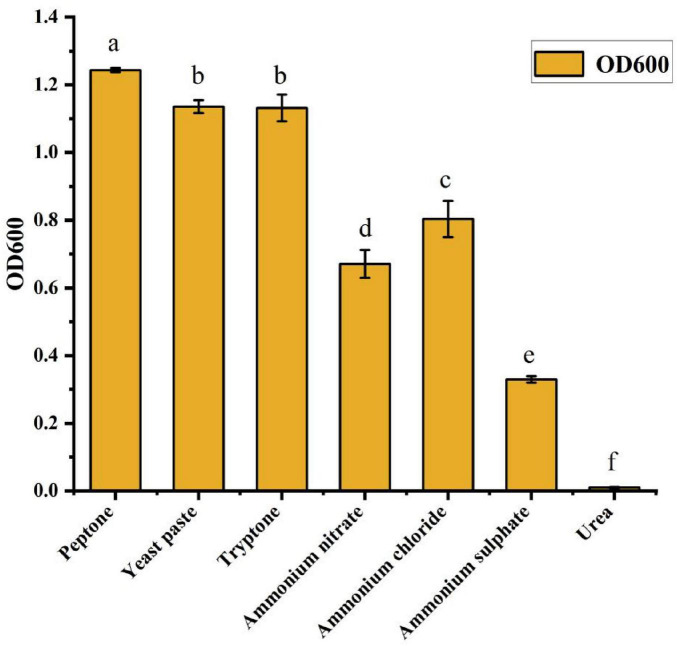
Effects of different nitrogen sources on the growth of *Bacillus amyloliquefaciens* ck-05. Bacterial growth was assessed by measuring optical density (OD) at 600 nm after 48 h of incubation in Luria-Bertan (LB) medium supplemented with various nitrogen sources (e.g., ammonium sulfate, urea, peptone, and yeast extract). Statistical analysis was performed using one-way analysis of variance (ANOVA), with lowercase letters (e.g., “a,” “b,” “c,” “d,” “e,” “f”) indicating significant differences (*p* < 0.05) between treatments, as determined by Tukey’s *post-hoc* test. Error bars represent standard deviation.

To optimize the nitrogen source for the fermentation culture of strain ck-05, various nitrogen sources were evaluated for their effects on cell growth, as indicated by the optical density at 600 nm (OD600). The nitrogen sources tested included peptone, yeast extract, tryptone, ammonium chloride, ammonium nitrate, ammonium sulfate, and urea. As shown in Figure 3, nitrogen sources had varying effects on the cell concentration in the fermentation broth. The OD600 values followed the order: peptone > yeast extract > tryptone > ammonium chloride > ammonium nitrate > ammonium sulfate > urea. Among the nitrogen sources, peptone demonstrated the most significant positive effect on cell growth. When peptone was used, the OD600 value of the fermentation broth was substantially higher than that observed with the other nitrogen sources, making it the most effective option for promoting cell density. Based on these results, peptone was selected as the optimal nitrogen source for the ck-05 fermentation medium. To identify the ideal concentration of peptone, a series of experiments was conducted with varying concentrations (5, 10, 15, 20, 25, and 30 g/L) added to the basic medium. The OD600 values of the fermentation broths were recorded and are summarized in Table 2. The data indicated that while the OD600 value did not show significant variation within the 10–30 g/L range, these treatments consistently resulted in higher OD600 values compared to the 5 g/L treatment. This suggests that a peptone concentration of at least 10 g/L is necessary to achieve optimal cell growth, and further increases beyond this threshold do not lead to significant improvement in cell density. Future work may focus on investigating the synergistic effects of peptone with other medium components or examining its impact on product yield and quality.

**TABLE 2:** Effect of different concentrations of peptone on the growth of strain ck-05, measured by OD600.

Concentration	5 g/L	10 g/L	15 g/L	20 g/L	25 g/L	30 g/L
OD600	1.02 ± 0.05b	1.26 ± 0.01a	1.23 ± 0.02a	1.26 ± 0.01a	1.26 ± 0.01a	1.24 ± 0.01a

Values represent the mean ± standard deviation (*n* = 3) of triplicate measurements. Different letters (a, b) indicate statistically significant differences between peptone concentrations (*p* < 0.05), based on a one-way analysis of variance (ANOVA).

#### 3.2.3 Screening of inorganic salt composition and concentration

**FIGURE 4:**
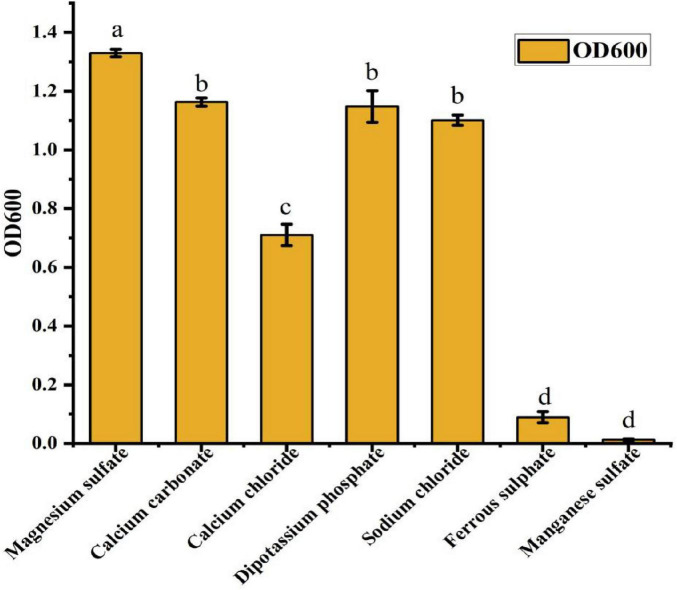
Effect of different inorganic salts on the growth of strain ck-05. Growth was assessed by OD600 at different salt concentrations. Data are presented as the mean ± standard deviation (*n* = 3), with different letters above the bars indicating significant differences (*p* < 0.05) as determined by one-way analysis of variance (ANOVA) analysis.

The selection of an appropriate inorganic salt is essential for optimizing the fermentation culture of strain ck-05. Various inorganic salts were tested to evaluate their effects on cell growth, as measured by the optical density at 600 nm (OD600). The inorganic salts included magnesium sulfate, dipotassium hydrogen phosphate, calcium carbonate, sodium chloride, calcium chloride, ferrous sulfate, and manganese sulfate. As illustrated in Figure 4, the OD600 values for these treatments followed the order: magnesium sulfate > dipotassium hydrogen phosphate > calcium carbonate > sodium chloride > calcium chloride > ferrous sulfate > manganese sulfate. Among these, magnesium sulfate exhibited the most significant enhancement of cell concentration, with an OD600 value that was markedly higher than those of the other tested salts. This superior performance led to the selection of magnesium sulfate as the optimal inorganic salt for the ck-05 fermentation medium. To determine the optimal concentration of magnesium sulfate, experiments were conducted using different concentrations (2.5, 3.0, 3.5, 4.0, 4.5, and 5.0 g/L). The OD600 values of the fermentation broths for these treatments are presented in Table 3. The results showed that while the OD600 values for concentrations in the 3.0–5.0 g/L range did not exhibit significant differences, they were consistently and significantly higher than the OD600 value recorded for the 2.5 g/L treatment. These findings suggest that a magnesium sulfate concentration of at least 3.0 g/L is required to support optimal cell growth, with no significant advantage in increasing the concentration beyond this level. This study underscores the critical influence of inorganic salt composition and concentration on the fermentation performance of strain ck-05. Magnesium sulfate was identified as a key component for promoting robust cell growth, and its optimal concentration was determined to be within the range of 3.0–5.0 g/L.

**TABLE 3:** Effect of different concentrations of magnesium sulfate on strain ck-05.

Concentration	2.5 g/L	3.0 g/L	3.5 g/L	4.0 g/L	4.5 g/L	5.0 g/L
OD600	1.02 ± 0.05b	1.26 ± 0.01a	1.23 ± 0.02a	1.26 ± 0.01a	1.26 ± 0.01a	1.24 ± 0.01a

Effect of magnesium sulfate concentrations on strain ck-05 growth, measured by OD600. Data are mean ± SD (*n* = 3). Different letters indicate significant differences (*p* < 0.05).

#### 3.2.4 Optimization of different culture conditions

Various culture conditions influenced the cell density of the CK-05 fermentation broth differently. The results are discussed below in terms of relative growth changes, with OD600 values used as a quantitative measure of bacterial growth.

**FIGURE 5:**
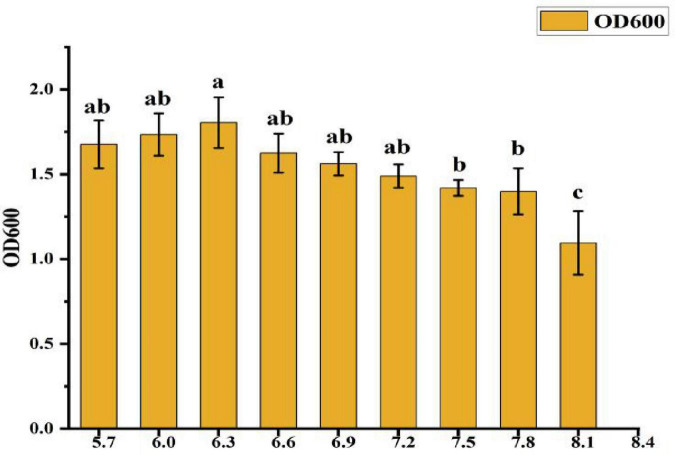
Effect of pH on strain ck-05 growth (OD600). Data are mean ± SD (*n* = 3). Different letters indicate significant differences (*p* < 0.05).

**FIGURE 6:**
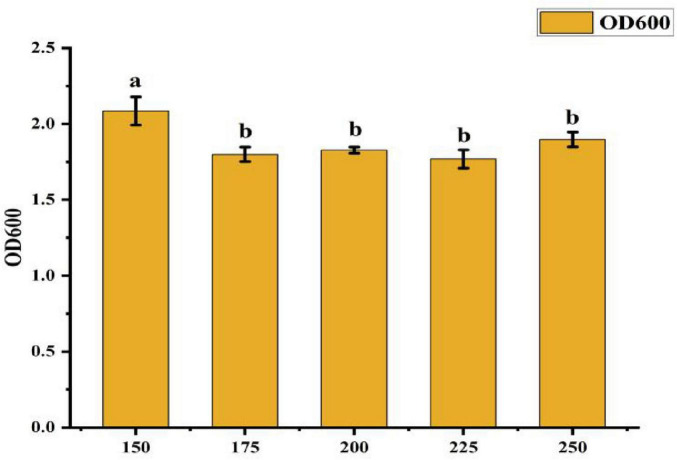
Effect of different rotate speeds on strain ck-05. Growth was assessed under varying rotation speeds to determine optimal conditions for strain CK-05. Data are presented as mean ± standard deviation (*n = 3*).

**FIGURE 7:**
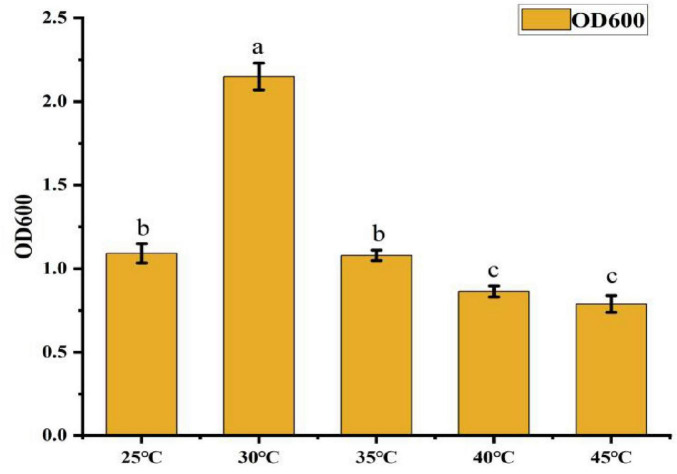
Effect of different temperatures on strain ck-05. Effect of different temperatures on the growth of strain CK-05. Growth was evaluated under varying temperature conditions to determine the optimal temperature for strain CK-05. Data are presented as mean ± standard deviation (*n = 3*).

**FIGURE 8:**
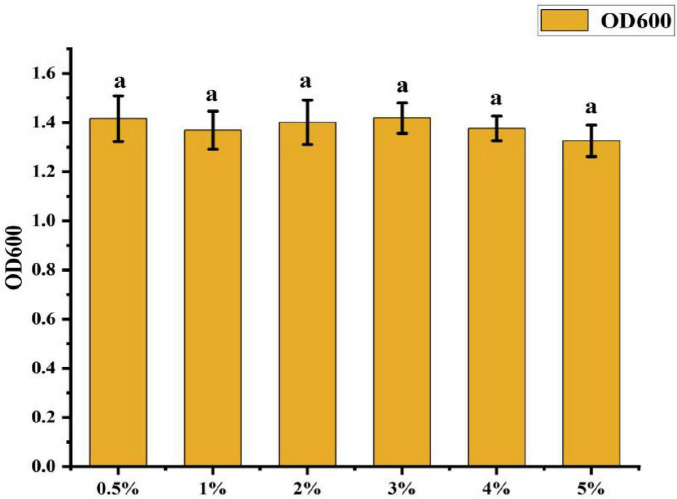
Effect of different inoculation amounts on strain ck-05. Growth was assessed under varying inoculation amounts to determine the optimal inoculum concentration for strain CK-05. Data are presented as mean ± standard deviation (*n = 3*).

**FIGURE 9:**
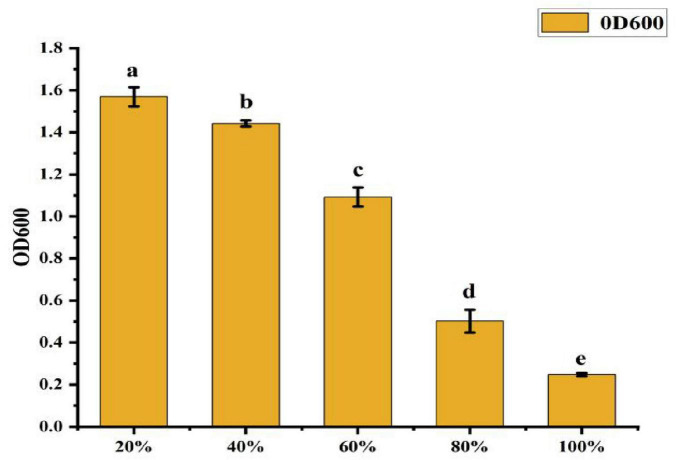
Effect of different bottling amounts on strain ck-05. Growth was measured under varying bottling volumes to determine optimal conditions for strain CK-05. Data are presented as mean ± standard deviation (*n = 3*).

• Effect of pH: As shown in Figure 5, the OD600 value reached 1.804 at a pH of 6.3, which was significantly higher than in other treatment groups. This represents a 65.5% increase in growth compared to the lowest OD600 value (1.09) observed at pH 8.1. The OD600 value increased as the pH rose from 5.7 to 6.3, but declined when the pH exceeded 6.3, indicating that pH 6.3 is optimal for CK-05 growth.

• Effect of Rotation Speed: Figure 6 indicates that the OD600 value was highest at 2.085 when the rotation speed was 150 r/min, which was significantly greater than in other treatments. This represents a 15.2% increase in growth compared to the next best rotation speed. No significant differences were observed among the other rotation speed treatments, suggesting that 150 r/min is the optimal condition for maximizing bacterial growth.

• Effect of Temperature: As shown in Figure 7, the OD600 value peaked at 2.15 when the temperature was 30°C, significantly higher than in other treatment groups. This represents a 172.8% increase in growth compared to the lowest OD600 value (0.788) observed at 45°C. When the temperature exceeded 35°C, the OD600 value gradually declined, indicating that 30°C is the optimal temperature for CK-05 growth.

• Effect of Inoculum Amount: Figure 8 shows that the OD600 value was highest at 0.5% inoculum amount, slightly higher than other treatments. However, the differences among treatments were not statistically significant, suggesting that inoculum amount had minimal impact on the cell density of CK-05 fermentation broth.

• Effect of Bottling Volume: As shown in Figure 9, the OD600 value was highest at 20% bottling volume, which was significantly greater than in other treatments. This represents a 769.2% increase in growth compared to the lowest OD600 value (0.247) observed at 100% bottling volume. As bottling volume increased, the OD600 value decreased significantly, indicating that 20% bottling volume is optimal for CK-05 growth.

The superior growth observed at lower bottling volumes can be attributed to several factors:

λ Enhanced Oxygen Availability: A 20% bottling volume leaves ample headspace, ensuring sufficient oxygen transfer for aerobic respiration, which is critical for the growth of Bacillus amyloliquefaciens CK-05.

λ Improved Agitation Efficiency: Lower volumes allow for better mixing and nutrient distribution, ensuring uniform access to nutrients throughout the medium.

λ Efficient Gas Exchange: The increased headspace facilitates the removal of metabolic byproducts (e.g., CO2) and the uptake of oxygen, creating a more favorable growth environment.

λ Reduced Shear Stress: Lower volumes minimize shear stress during shaking, preventing potential damage to bacterial cells and supporting optimal growth.

In contrast, higher bottling volumes (e.g., 100%) limit oxygen availability, restrict agitation efficiency, hinder gas exchange, and increase shear stress, all of which contribute to reduced bacterial growth. These findings highlight the importance of optimizing bottling volume to maximize the growth of strain CK-05 in fermentation cultures.

### 3.3 Response surface optimization

#### 3.3.1 Plackett-Burman design experiment

The Plackett-Burman design experiment was used to screen eight factors affecting the fermentation conditions of strain ck-05 (Zhang et al., 2022), including soluble starch (A), peptone (B), magnesium sulfate (C), pH (D), rotational speed (E), temperature (F), inoculation amount (G), and bottling volume (H). Each factor was set at two levels: high (+) and low (−). The levels for each factor are shown in Table 4. The OD600 value after fermentation of strain ck-05 was used as the response variable. A total of 12 groups of experiments were designed, with three replicates per group. The results are summarized in Table 5.

**TABLE 4:** Factors and levels of Plackett-Burman (PB) experimental design.

Run no.	Variable	Level	
		**−1**	**1**
A	Soluble starch	15 g/L	30 g/L
B	Peptone	5 g/L	15 g/L
C	Magnesium sulfate	2.5 g/L	4.0 g/L
D	pH	6.4	8.2
E	Rotate speed	150 r/min	225 r/min
F	Temperature	30°C	45°C
G	Inoculation amount	0.5%	1%
H	Bottled volume	20%	100%
I	Empty items	–	–
J	Empty items	–	–

**TABLE 5:** Design and corresponding results of Plackett-Burman (PB) experiment.

Run no.	Variable								OD600
	**Soluble starch**	**Peptone**	**Magnesium sulfate**	**pH**	**Rotate speed**	**Temperature**	**Inoculation amount**	**Bottled volume**	
1	−1	1	−1	1	1	−1	1	−1	2.53 ± 0.05
2	1	1	1	1	−1	−1	−1	1	0.46 ± 0.03
3	−1	−1	−1	1	1	1	1	1	0.24 ± 0.00
4	−1	1	1	−1	1	1	1	−1	2.10 ± 0.06
5	1	−1	1	−1	1	−1	−1	−1	2.09 ± 0.01
6	−1	1	−1	−1	−1	1	−1	−1	1.86 ± 0.02
7	1	−1	1	−1	−1	1	1	1	1.34 ± 0.01
8	−1	−1	1	1	−1	−1	1	−1	1.59 ± 0.01
9	1	−1	−1	1	−1	1	−1	−1	1.03 ± 0.01
10	1	1	−1	−1	−1	−1	1	1	0.35 ± 0.01
11	1	1	1	1	1	1	−1	1	0.29 ± 0.01
12	−1	−1	−1	−1	1	−1	−1	1	0.69 ± 0.15

**FIGURE 10:**
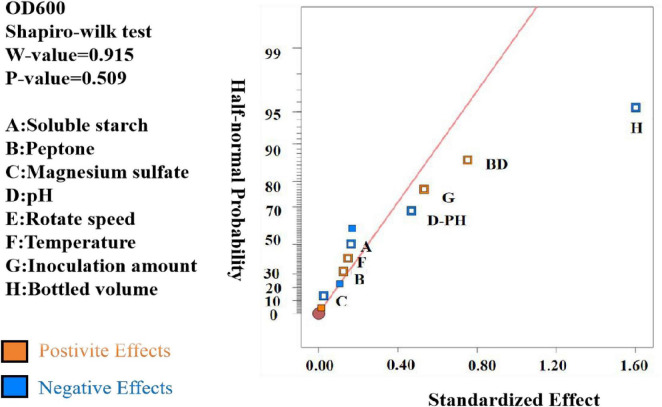
Semi-normal probabilistic plot of standardized effects (α = 0.05).

**FIGURE 11:**
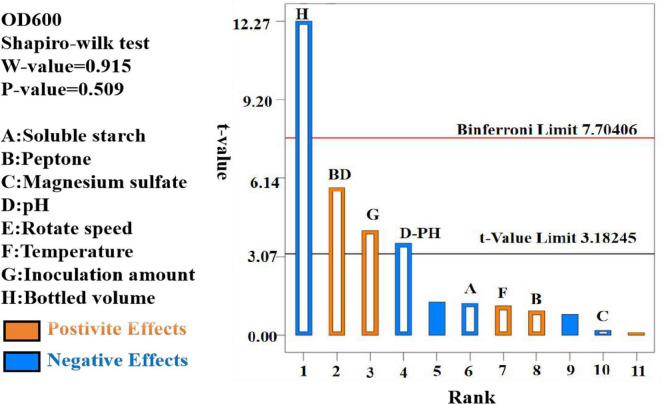
Pareto plot of standardized effects (α = 0.05).

The data were analyzed using Design Expert 13.0, resulting in a semi-normal probability effect diagram for standardized effects (Figure 10) and a Pareto chart of standardized effects (Figure 11). From Figure 10, the standardized effect points for factors H, G, and D deviated significantly from the fitting line, indicating that these factors were significant (*P* < 0.05). Thus, bottling volume, inoculation amount, and pH were identified as the significant factors influencing OD600. The standardized effect points for other factors were smaller and not significant. Figure 11 further confirmed the magnitude and importance of the effects, with factors H, G, and D exceeding the t-value threshold, verifying their significance.

Table 6 shows that the model’s *P*-value is 0.0106, which is less than 0.05, indicating statistical significance and reliability. The multiple correlation coefficient (R^2^) is 0.9860, suggesting a strong correlation. The adjusted determination coefficient (R^2^ adj) is 0.9488, indicating that 94.88% of the variability in the test data can be explained by this regression model. The coefficient of variation (CV), which reflects the reliability and precision of the experiment, is 9.55%. A CV value this low indicates high credibility and accuracy. Additionally, an adequate precision value above 4.0 is considered reasonable; in this case, the value reached 14.53, further supporting the test’s precision.

**TABLE 6:** Main effect analysis of Plackett-Burman (PB) experiments design.

Source	df	sum of squares	Mean square	F-value	*P*-value	Significant
Model	8	7.18	0.8979	26.49	0.0106	significant
A (soluble starch)	1	0.0534	0.0534	1.57	0.2984	–
B (peptone)	1	0.031	0.031	0.915	0.4094	–
C (magnesium sulfate)	1	0.0014	0.0014	0.0401	0.8541	–
D (pH)	1	0.437	0.437	12.89	0.037	significant
E (rotate speed)	1	0.044	0.044	1.3	0.3373	–
F (temperature)	1	0.567	0.567	16.73	0.0564	–
G (inoculation amount)	1	5.1	5.1	150.61	0.0012	significant
H (bottled volume)	1	1.13	1.13	33.31	0.0103	significant
Residual	1	0.1017	0.0339	–	–	–
Total	11	7.28	–	–	–	–
R^2^	0.9860	–	–	–	CV	9.55
R^2^_adj_	0.9488	–	–	–	Adeq Precisior	14.53

Through multiple regression fitting of the data, the following regression equation was obtained:

OD600 = 1.21–0.1253A + 0.0508B–0.0154C–0.1908D–0.0872E + 0.0838F + 0.2570G–0.7654H

#### 3.3.2 Steepest climbing test

The steepest climbing test was conducted based on the results of the Plackett-Burman (PB) test. Bottling volume, inoculation amount, and pH were identified as the three significant influencing factors. Among these, bottling volume and pH exhibited negative effects, while inoculation amount showed a positive effect. The step lengths and directions for these three factors were determined according to the PB test results. The experimental design and results are presented in Table 7, with each group repeated three times.

**TABLE 7:** Results of the steepest climb experiment.

Run no.	Bottled volume	Inoculation amount	pH	OD600
1	60%	0.4%	7.5	1.91 ± 0.12
2	50%	0.5%	7.2	2.01 ± 0.09
3	40%	0.6%	6.9	2.35 ± 0.03
4	30%	0.7%	6.6	2.52 ± 0.04
5	20%	0.8%	6.3	2.49 ± 0.03
6	10%	0.9%	6.0	2.46 ± 0.05

Based on the single-factor experiment results, the fixed values for other factors were as follows: soluble starch, 15 g; peptone, 15 g; magnesium sulfate, 4.0 g; rotational speed, 150 r/min; and temperature, 30°C.

Based on the results of the steepest climbing test, the response value (OD600) for treatment 4 was the highest. Therefore, treatment 4 was selected as the central point for the response surface test, with the following conditions: bottling volume of 30%, inoculation amount of 0.7%, and pH of 6.6.

#### 3.3.3 Box-Behnken design

Based on the results from the steepest climbing test, a Box-Behnken design was implemented with treatment 4 as the central point. Three factors—bottling quantity, inoculation amount, and pH—were selected as independent variables, and a three-factor, three-level test was established using the Box-Behnken design, with OD600 as the response variable. A total of 17 experimental trials were designed, with each trial repeated three times. The results are shown in Table 8.

**TABLE 8:** Design and results of Box-Behnken test.

Run no.	Bottled volume	Inoculation amount	pH	OD600
1	0	0	0	2.54 ± 0.23
2	1	1	0	2.34 ± 0.25
3	0	0	0	2.51 ± 0.24
4	0	0	0	2.49 ± 0.19
5	1	0	−1	2.31 ± 0.25
6	0	0	0	2.53 ± 0.19
7	0	−1	−1	1.78 ± 0.08
8	0	1	−1	1.86 ± 0.13
9	0	1	1	1.93 ± 0.24
10	−1	1	0	2.12 ± 0.23
11	1	−1	0	2.33 ± 0.21
12	−1	0	1	1.96 ± 0.18
13	1	0	1	2.35 ± 0.24
14	0	0	0	2.52 ± 0.15
15	−1	−1	0	1.79 ± 0.14
16	−1	0	−1	1.75 ± 0.16
17	0	−1	1	2.12 ± 0.13

Through quadratic multiple regression analysis of the data, the following quadratic polynomial equation was obtained (2):

OD600 = 2.52 + 0.2138A + 0.0287B–0.0825C–0.0800AB + 0.0425AC + 0.0675BC–0.1015A^2^–0.2715B^2^–0.3240C^2^.

The model’s correlation coefficient (R^2^) is 0.9750, indicating a good fit between the model and the actual data, and the test results can be analyzed using this equation. The analysis of variance (ANOVA) for the response surface experiments is shown in Table 9.

**TABLE 9:** Analysis of variance of regression model.

Source	df	sum of squares	Mean square	*F*-value	*P*-value	Significant
Model	9	1.35	0.1498	30.38	< 0.0001	Significant
A-bottled volume	1	0.3655	0.3655	74.15	< 0.0001	Significant
B-inoculation amount	1	0.0066	0.0066	1.34	0.2848	–
C-pH	1	0.0544	0.0544	11.05	0.0127	–
AB	1	0.0256	0.0256	5.19	0.0567	–
AC	1	0.0072	0.0072	1.47	0.2653	–
BC	1	0.0182	0.0182	3.70	0.0959	–
A^2^	1	0.0434	0.0434	8.80	0.0209	Significant
B^2^	1	0.3104	0.3104	62.96	< 0.0001	Significant
C^2^	1	0.4420	0.4420	89.67	< 0.0001	Significant
Residual	7	0.0345	0.0049	–	–	–
Lack of fit	3	0.0330	0.0110	29.75	0.0034	Not significant
R^2^	0.9750	–	–	–	CV%	3.21
R^2^_*adj*_	0.9429	–	–	–	Adeq Precisior	14.378

From Table 9, it is evident that the model is highly significant (*P* < 0.0001), while the lack-of-fit term is not significant (*P* > 0.05). The model’s R^2^ is 0.99750, and the adjusted R^2^ is 0.9429, indicating that the model fits well. The model can explain 94.29% of the variability in the response values. This model can be used to analyze and predict the OD600 value of strain ck-05.

The results show that the primary factor A (bottling quantity) has a highly significant effect on OD600, while B (inoculation amount) and C (pH) have significant effects. The interaction terms AB, AC, and BC are not significant, but the quadratic terms A^2^, B^2^, and C^2^ are significant. The relationship between the factors and the response value is not a simple linear relationship, with the factors influencing the response in the following order: A > C > B, meaning bottling quantity > pH > inoculation amount.

**FIGURE 12:**
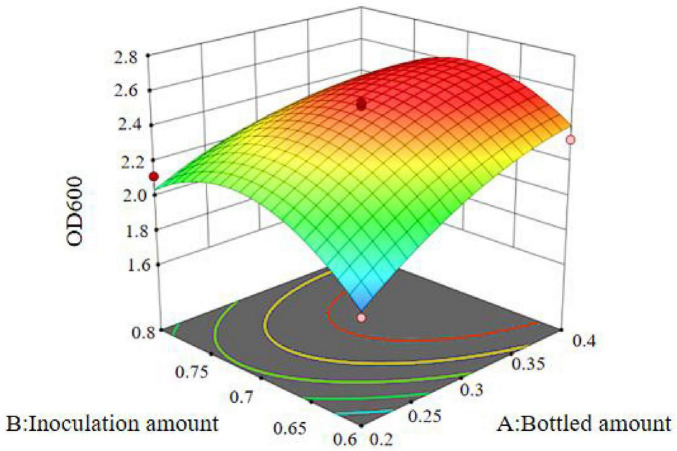
Response surface of interaction between bottled amount and inoculation amount on OD600.

**FIGURE 13:**
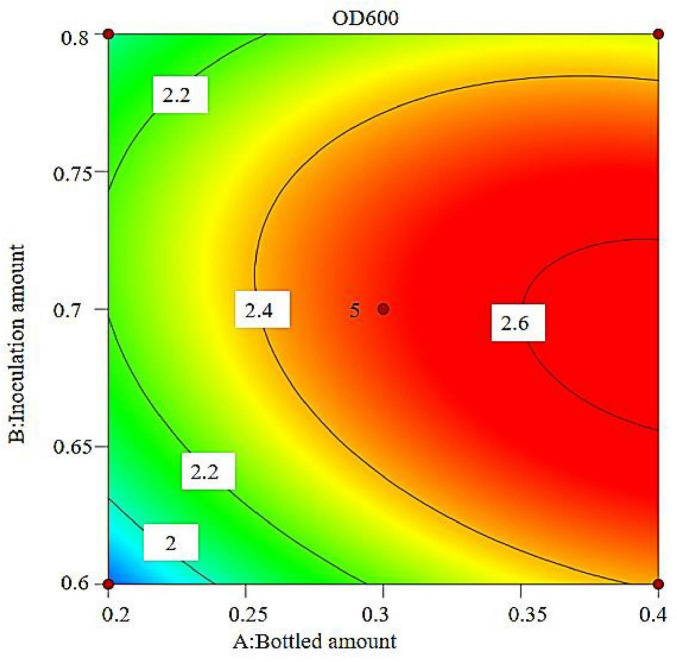
Contour lines of interaction between bottled amount and inoculation amount on OD600.

**FIGURE 14:**
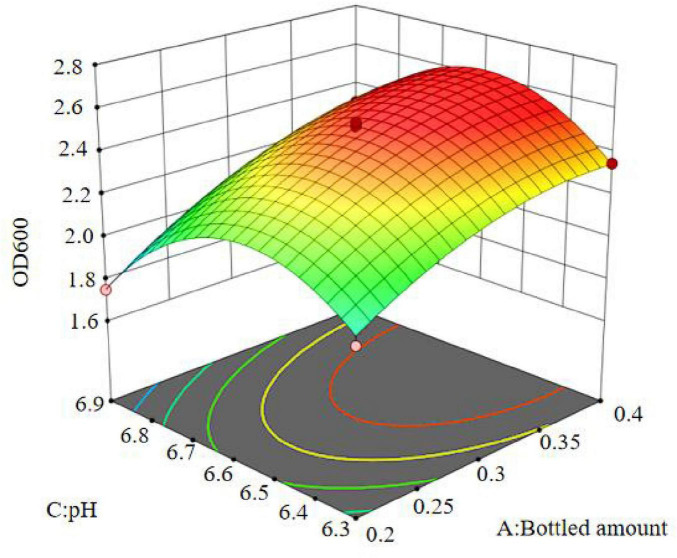
Response surface of interaction between bottled amount and pH on OD600.

**FIGURE 15:**
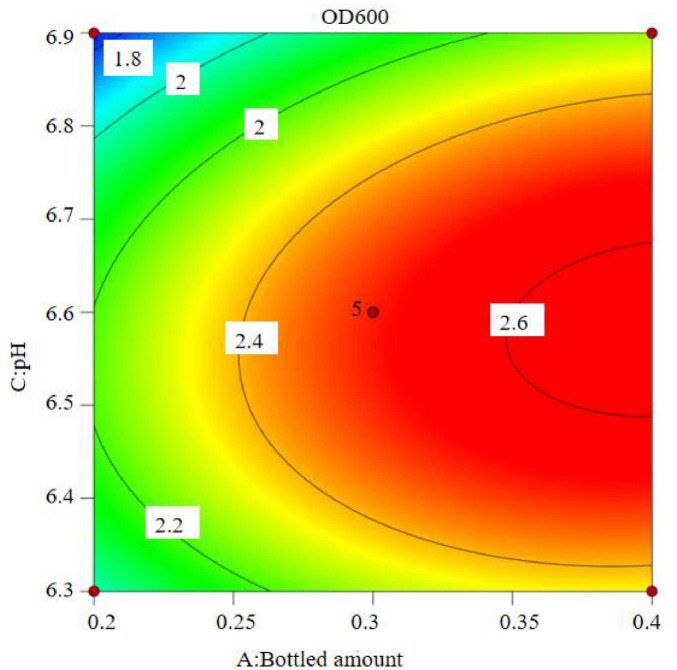
Contour lines of interaction between bottled amount and pH on OD600.

**FIGURE 16:**
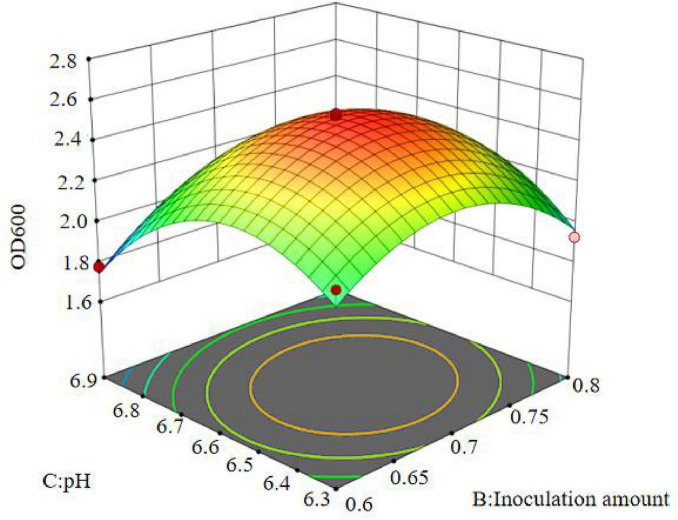
Response surface of interaction between inoculation amount and pH on OD600.

**FIGURE 17:**
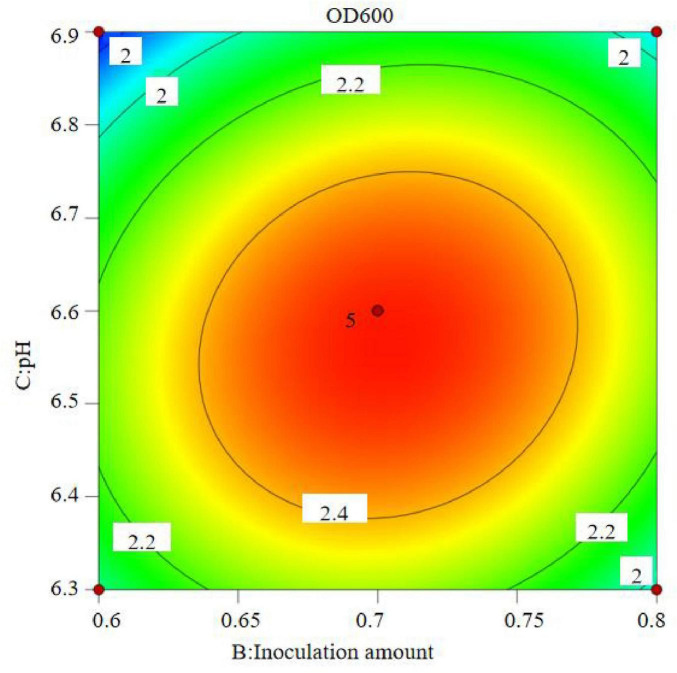
Contour lines of interaction between inoculation amount and pH on OD600.

The regression equation was used to generate the response surface analysis diagrams in Design Expert, as shown in Figures 12–17. The 3D response surface plots and contour plots clearly show that the experiments included the area where the maximum value is located.

Based on the model’s analysis and prediction in Design Expert, the optimal conditions were found to be a bottling quantity of 40%, an inoculation amount of 0.8%, and a pH of 6.6. Under these conditions, the OD600 value reached its maximum at 2.35.

#### 3.3.4 Confirmatory test

A confirmatory test was conducted under the optimized conditions, and the OD600 value was found to be 2.35. The experimental values matched the theoretical values, indicating that the modified model can accurately predict the fermentation conditions for strain ck-05. This also confirms the feasibility of optimizing the fermentation and cultivation conditions of strain ck-05 using the response surface method.

## 4 Discussion

Response surface analysis uses statistical and mathematical models to analyze and optimize multi-factor systems. The Box-Behnken experimental design is a commonly used model for optimizing media and culture conditions in biological fermentation processes. This method not only allows for the establishment of surface models and fitting equations for continuous variables, but also evaluates the various factors and their interactions that affect microbial growth and fermentation. It helps determine the optimal range of conditions, yielding more accurate and effective results with fewer experimental groups, thereby saving resources, reducing costs, and maximizing efficiency.

The optimization of culture media is a crucial step in the industrial production of microbial preparations, as different strains have specific preferences for nutrient components in the medium (Luo, 2010). When optimizing medium composition, the focus is primarily on the carbon source, nitrogen source, and inorganic salts (Asma et al., 2017). Selecting appropriate carbon and nitrogen sources can significantly promote microbial growth and reproduction, improving the quality and yield of metabolites. Based on findings from previous research, glucose, sucrose, fructose, lactose, mannitol, soluble starch, and maltose were chosen as potential carbon sources. The results from single-factor tests revealed that soluble starch resulted in the highest OD600 value, which aligned with the findings of other researchers (Li et al., 2019). After identifying the optimal carbon source, nitrogen sources such as peptone, yeast extract, tryptone, urea, ammonium nitrate, ammonium chloride, and ammonium sulfate were selected. The results showed that peptone yielded the highest OD600 value, making it the best nitrogen source, consistent with findings from other studies (Liu et al., 2023). For inorganic salt screening, magnesium sulfate, calcium carbonate, ferrous sulfate, dipotassium hydrogen phosphate, sodium chloride, manganese sulfate, and calcium chloride were tested. The results indicated that magnesium sulfate produced the highest OD600 value in the fermentation broth, with 4 g/L identified as the optimal concentration. In a similar study, the fermentation medium of *Bacillus subtilis* NHS1 was optimized using response surface methodology, improving the optimization effect by 1.5 times compared to previous methods (Hu et al., 2018).

Culture conditions play a crucial role in bacterial reproduction (Wang et al., 2017). Changes in bottling quantity affect the dissolved oxygen content in the culture medium. Initially, increasing the bottling quantity causes the cell concentration to rise, but it eventually decreases. When the bottling quantity is small, the dissolved oxygen in the culture medium is sufficient, but nutrients become limited for bacterial growth. Prolonged culture also leads to a gradual decrease in moisture, negatively affecting bacterial cell growth and resulting in a lower final concentration of bacterial broth. As the bottling volume increases, the dissolved oxygen coefficient gradually decreases, and cell hypoxia can inhibit growth. Therefore, adjusting the balance between the dissolved oxygen coefficient and nutrients is crucial to determining the optimal bottling quantity. In this study, the optimal bottling quantity was determined to be 40% of the bottle’s volume. Some studies have shown that bacteria are highly sensitive to temperature, with the optimal temperature for *Bacillus amyloliquequebilis* Z17-2 being 35°C (Song et al., 2021). However, the results of this study indicated that the optimal culture temperature for strain ck-05 is 30°C. The appropriate shaking speed on a shaking table can influence the dissolved oxygen content in the culture medium and promote bacterial growth and reproduction. For *Pseudomonas aeruginosa* TCd-1, the best shaking speed was found to be 160 r/min (Wang et al., 2020). In this study, the optimal shaking speed for strain ck-05 was 150 r/min. Additionally, the initial pH of the culture medium significantly impacts bacterial growth. pH may influence the permeability of cell membranes, thereby affecting bacterial growth. This study determined that the optimal pH value for strain ck-05 is 6.6.

## 5 Conclusion

This study systematically optimized the fermentation and cultivation conditions for strain ck-05 using response surface methodology (RSM) combined with the Box-Behnken experimental design. This rigorous statistical approach enabled the precise identification of key factors affecting fermentation and the determination of their optimal levels to maximize cell growth. Soluble starch, peptone, and magnesium sulfate were identified as the optimal carbon source, nitrogen source, and inorganic salt, respectively. Further refinement of culture parameters, including temperature, pH, shaking speed, fermentation duration, and bottling capacity, established the following optimal conditions: 15 g/L soluble starch, 15 g/L peptone, 4.0 g/L magnesium sulfate, 30°C incubation temperature, 40 h fermentation duration, 40% bottling capacity, 0.8% inoculum size, and an initial pH of 6.6. Under these optimized conditions, the OD600 value of the fermentation broth increased from 1.36 to 2.35, representing a 72.79% improvement in cell concentration. The study highlights the effectiveness of RSM and the Box-Behnken design in optimizing biological fermentation processes, achieving significant improvements in fermentation efficiency while reducing experimental costs. The findings provide a strong theoretical foundation for the industrial application of strain ck-05, particularly as a microbial fertilizer. Future research should focus on scaling up the fermentation process and assessing the field performance and environmental impact of strain ck-05 in agricultural systems.

## Data Availability

The original contributions presented in this study are included in this article/supplementary material, further inquiries can be directed to the corresponding authors.
